# Distribution of the Order Lampriformes in the Mediterranean Sea with Notes on Their Biology, Morphology, and Taxonomy

**DOI:** 10.3390/biology11101534

**Published:** 2022-10-19

**Authors:** Marco Albano, Claudio D’Iglio, Nunziacarla Spanò, Jorge Manuel de Oliveira Fernandes, Serena Savoca, Gioele Capillo

**Affiliations:** 1 Department of Chemical, Biological, Pharmaceutical and Environmental Sciences, University of Messina, 98166 Messina, Italy; 2 Institute for Marine Biological Resources and Biotechnology (IRBIM), National Research Council (CNR), Section of Messina, 98100 Messina, Italy; 3 Department of Biomedical, Dental and Morphological and Functional Imaging, University of Messina, Via Consolare Valeria 1, 98125 Messina, Italy; 4 Faculty of Biosciences and Aquaculture, Nord University, 8049 Bodø, Norway; 5 Department of Veterinary Sciences, University of Messina, 98168 Messina, Italy

**Keywords:** zoological records, teleost, deep-sea fishes, phylogeny, marine environment, ontogeny, by-catch, biodiversity, biomonitoring, Acanthomorpha

## Abstract

**Simple Summary:**

The distribution of teleost fishes is of fundamental importance to comprehending how these species contribute to the ecological equilibria of marine areas. In these terms, fishes of the order Lampriformes are circumglobally distributed, and are characterized by a peculiar morphology. Information on this fish group is scarce to absent regarding both their biological and distribution features, especially for the Mediterranean Sea. Moreover, the systematics of the group underwent several rearrangements, and molecular support is still scarce. This review aimed to describe the biological and ecological characteristics of the order Lampriformes, and to compile the scattered Lampriformes records from the literature. Lampriformes represent a taxon of high biological and ecological importance whose knowledge is limited by their low commercial value and rarity. The bathymetrical range of distribution also makes these species very interesting from an adaptive point of view. Four families are present in the Mediterranean Sea—Lampridae, Lophotidae, Regalecidae, and Trachipteridae—with a total of six species. Despite the scarce information on this taxon, the present review, which collected all reports of the Mediterranean Sea, represents a baseline to increase the knowledge of these rare and important species, and to encourage new studies in this area. Moreover, a phylogenetic reconstruction of Lampriformes based on the cytochrome oxidase subunit 1 (mt-COI) gene sequences questions the current evolutionary relationships between some species.

**Abstract:**

Lampriformes are circumglobally distributed and contain several families of strictly marine bony fishes that have a peculiar morphology. Lampriformes systematics is affected by limitations in biometric, meristic, and molecular data; for this reason, it underwent several rearrangements in the past. This review aimed to describe the biological and ecological characteristics of the order Lampriformes, summarizing the current taxonomy of the group. The main aim was to clarify what is known about the distribution of the order Lampriformes in the Mediterranean Sea, collecting all the scarce and fragmented reports and notes on their occurrence. Knowledge scarcity is due to their solitary nature, in addition to their low to absent economic value. Despite this, the order Lampriformes represents a taxon of high biological and ecological importance. The high depth range of distribution characterizes their lifestyle. In the Mediterranean Sea, four families are present—Lampridae, Lophotidae, Regalecidae, and Trachipteridae—with the following species respectively, *Lampris guttatus* (Brünnich, 1788), *Lophotus lacepede* (Giorna, 1809), *Regalecus glesne* (Ascanius, 1772), *Trachipterus arcticus* (Brünnich, 1788), *T. trachypterus* (Gmelin, 1789), and *Zu cristatus* (Bonelli, 1819). Data deficiencies affect information on this taxon; the present review, which collected all the reports of the Mediterranean Sea, creates a baseline for depicting the biogeography of these rare and important species.

## 1. Introduction

The order Lampriformes is a globally distributed taxon that contains several families of strictly marine bony fishes with a peculiar morphology [1,2]. Despite their wide distribution, they are not commonly reported due to their rarity and life cycle that result in their elusiveness [3,4]. The scarcity of reports in the literature results in little information regarding their presence and distribution. Indeed, even if they are biologically interesting species, they represent fishing waste from a commercial point of view. Therefore, they are not retained and reported, leading to a significant gap in data collection [5]. Their morphological aspects led to characteristic common names of these species (ribbonfish, velifers, tube-eye, crestfish, oarfish, dealfish, tapertails, unicornfish, and inkfish). Due to the distinctive shape of most of these species, jointly with their iridescent colors, these fishes became historically involved in several legends and popular myths [4]. Moreover, the larval stages of these species are often very different from the adult stages, both in morphology and lifestyle aspects [6]. All these factors contribute to the interest of zoological researchers and public curiosity.

The research interest in this order is linked to the ecological role of lampriform species that are involved in many bathypelagic food webs interactions. Moreover, during evolution, deep-sea species have developed highly specialized features to inhabit deep-sea environments, such as the maintenance of a vertical position in the water column during predation, which was reported also for some Lampriformes species [7,8]. This feature may help these fish in the visualization of prey against downwelling light, and, at the same time, allow these elongate fish to minimize their appearance [9]. However, studies that are related to adaptations of Lampriformes species to deep-sea life are scarce and, in consideration of their morphological features, require further in-depth investigations, especially from functional and genetic points of view [10,11]. Studying the adaptation to deep-sea life is essential both from an anatomical and physiological point of view, in relation to their ecological features [12]. Moreover, this group is quite important from a phylogenetic point of view, due to their ancient origins among teleosts [13,14].

Lampriformes systematics is affected by limitations in biometric, meristic, and molecular data; for this reason, it underwent several rearrangements in the past. Some researchers have included other deep-sea fish families (e.g., Ateleopodidae, Megalomycteridae, Mirapinnidae, and Stylephoridae) based on morphological identifications of the group. Still, recent phylogenetic analyses, which were also based on molecular approaches, excluded these additional families that are currently considered unrelated [15,16]. However, this aspect is under continuous debate, and few phylogenetic studies of species-level relationships have been carried out on Lampriformes. Hence, to better assess the phylogenetic relationship of the group, more in-depth molecular data on families, genera, and species are required.

Environmental evolution in global marine ecosystems leads to continuous rearrangement of faunal stands [17]. In recent years, it is known that global climate changes have contributed to modifying several important environmental parameters of aquatic ecosystems, such as salinity, temperature, and pH (for the strong influence on dissolved CO_2_) [18,19]. Currently, the scientific community is working on understanding how these changes could affect aquatic organisms by trying to develop further predictive models [20,21]. The influences on marine faunal assemblages are various, and not only attributable to climate change. It is known that reproductive behaviors, habitat preferences, feeding behaviors, genetic adaptation, and especially anthropic pressure (with its double impacts of fishing and pollution) [22,23], lead to complex biocenotics dynamics. These cannot be explained without considering all these factors that impact the species. Biological monitoring assumes a pivotal role, through maintaining records of previously undiscovered species, new records of invasive alien species, and rare or uncommon, rarely reported ones [24,25,26].

Summarizing how pelagic organisms such as many teleosts are distributed is essential to understanding how they face environmental modifications or biological interactions. Indeed, phenomena such as areal rearrangement can lead the species to different fates, depending mainly on their ecological plasticity [27]. Fishes have two different ways of dispersing in the marine environment, geographically and bathymetrically [28,29]. Bathymetrical dispersion especially requires specific adaptations by the organisms to be realized, which mainly involve the sense organs and other structures. Indeed, in moving from shallow to deep environments, fishes encounter high illumination differences that drive them to make visual adaptations [9,30]. An increase in depth also influences structural hearing adaptations in fish that usually live under increased atmospheric pressure [31]. Not secondary are the modifications that occur in several species due to altered pressure in the gills [32] and the muscles [33,34], or in responding to oxygen limitation [35,36].

The Mediterranean Sea covers about 2,000,000 km^2^ on the European and the African continents [23]. It has a western communication with the Atlantic Ocean via the Strait of Gibraltar, and an eastern one with the Black Sea via the Dardanelles and the Bosphorus [37]. The recorded maximum depth of the basin was 5093 m, while the mean depth was about 1500 m; however, due to its fragmented nature, significant differences exist [26,38]. Indeed, the Mediterranean Sea is divided into several sub-basins that are variously connected and separated from one another by different geological elements, such as thresholds, peninsulas, or islands, of which the basin is rich in [39,40,41].

The biodiversity of Mediterranean fishes has been continuously evolving in the last decades because of non-indigenous species invasions, and the influence of described climate change effects [42,43,44]. Monitoring the occurrence and distribution of fish species is of fundamental importance, especially in semi-closed basins such as the Mediterranean Sea. The peculiar morphology of this basin divides it into several biologically different areas, which leads to enormous difficulties in carrying out research surveys that can cover it entirely [45]. Moreover, several fish species are difficult to detect and correctly identify during commercial fishing activities. Indeed, biologically interesting species often represent fishing waste and therefore, are not reported, leading to significant gaps in data collection [46,47]. Only *Lampris guttatus* (Brünnich, 1788) is appreciated and prized as by-catch in some areas [48]. The rest of the lampriform species are considered inedible; hence, they are discarded into the sea immediately after their capture [2].

This review aimed to describe the biological and ecological characteristics of the order Lampriformes, resuming the current taxonomy of the group. The main aim was to clarify what is known about the order of Lampriformes in the Mediterranean Sea, compiling all the scarce and fragmented reports and notes on their occurrence. Better knowledge will enhance the scientific community’s attention on this important taxon. This review will lead to significant attention on the Lampriformes order during commercial fishing activity, and to experimental surveys that collect more data and enable the scientific community to deepen their knowledge of this crucial geographical area.

## 2. Biological and Ecological Features of Lampriformes

Morphologically, most Lampriformes are characterized by bright silvery colors and very colorful fins that are mainly shades of red [2,4]. Sometimes, the first ray of the dorsal fin is quite elongated and evident, characterizing the appearance as in the case of *Zu cristatus* (Bonelli, 1819) [49]. As members of Subphylum Vertebrata and Subclass Teleostei, lampriform fishes are characterized by a developed axial skeleton. The elongate form, such as that of trachipterids, has a highly variable number of vertebrae comprising between 60 and 150 [1,50]. In contrast, the most compressed body species possess fewer vertebrae (33 to 46), such as the case of lamprids. Notably, this order features quite different patterns of small bones and ligaments associated with their anterior vertebrae, which probably evolved as a result of their characteristic swimming behavior and deep-sea life [1,51,52]. Other structural differences with the common teleost are mainly found in the head structure, with its lack of a ligamentous link between the cheekbones and the upper jaws (maxillae), supported by the nasal cartilage placed in the frontal region of the skull in a hollow [1]. These peculiar features are significant from a functional point of view for lampriform species, because they enable the upper jaws to be stretched forward during feeding behavior.

This extreme jaw protrusion in some species permits the expansion of the mouth of up to 40 times in dimension during predation, allowing capture of even the most evasive of planktonic prey items in darkness. Indeed, lampriforms feed mainly on small Polychaeta, medium/small-sized pelagic cephalopods, and crustaceans, Malacostraca included [53,54]. Moreover, they prey on small fishes that are not easy to capture in the dark deep-sea environment [53,54]. Borme and Voltolina also reported the occurrence in the stomach of *Trachipterus trachypterus* of some macroalgae, such as *Ulva intestinalis* (Linnaeus, 1753) and *Cystoseira compressa* ((Esper) Gerloff and Nizamuddin, 1975); this shows the fascinating omnivorous, opportunistic nature of the species [54]. Regarding feeding interactions as prey, lampriform fishes have been sporadically found in the stomachs of cetaceans, such as the sperm whale (*Physeter macrocephalus,* Linnaeus, 1758), chondrichthyans such as blue shark (*Prionace glauca*, Linnaeus, 1758), or large-sized bony fish predators such as tunas [55,56]. Moreover, some typical enteric parasites of fishes, such as *Ascaris capsularia* (Rudolphi, 1802), *Scolex polymorphus*, (Rudolphi, 1819), and *Anisakis physeteris* (Baylis, 1923), have been reported in some Lampriformes species [57].

Information about the reproduction of Lampriformes is very scarce due to their elusive nature [58]. Even though spawning has never been observed, lampriform fishes are considered broadcast spawners because the eggs are planktonic, and several authors have used them to record the presence of some species [6,59]. The eggs have a diameter of about 2–6 mm and remind of the species’ coloration with their bright with reddish hues. At hatching, the larvae have fully developed mouths and digestive tracts, and begin to feed immediately on minute plankton [6]. The larval stages of lampriforms are beautiful and identifiable fishes characterized by their evident, ornamented dorsal and pelvic long fin rays. It is known that trachipterids undergo metamorphosis, passing from the larval to the juvenile form [60,61].

Moreover, from a habitat point of view, some species prefer shallow water during larval/juvenile stages, eventually moving to the deep-sea environment during adult life. These transitions from shallow, nearshore habitats, to the deep open ocean, is one of the two significant events that were hypothesized to characterize evolution of the order Lampriformes [13]. The family of Veliferidae is a moderate-sized coastal fish group that inhabits shallow waters for their entire life cycle. Some authors have reported finding it at a maximum depth of 110 m. It seems to be the most ancient group within the Lampriformes order [62]. In the adult stage, all other lampriforms are open-ocean, epipelagic, mesopelagic, or bathypelagic fishes. Some adult specimens of the Lampridae family were also detected in shallow waters near the surface, but, apart from these occasional events, it is not considered a coastal water group [4]. An essential event in the evolution of the lampriform lineage is represented by the colonization of deep-sea environments made by other families of the group as a result of the necessary functional adaptations [33,63]. The second significant evolutionary transition relates to body shape, which passes from oval-shaped and deep-bodied veliferids and lamprids, to elongate forms of oar-ribbon fishes (Regalecidae, Trachipteridae) [61].

As mentioned, knowledge about this taxon is scarce in relation to difficulties in finding and monitoring live specimens, which rarely occurs. This difficulty is also due to their solitary nature, confirmed by the records present in the literature and collected in this review manuscript, which mainly concern occurrences of single specimens, very rarely two or three [5]. Whereas the generalist fishing methods through which they are caught, such as through trawling nets or longline fisheries, the rare occurrence of one specimen highlights their solitary nature and already known rarity. Experimental studies are absent in the literature, and very little is known about the most suitable habitats of these species, their preferences or interactions in aquatic communities, and their role in deep-sea food webs. Occasional underwater surveys with ROVs, or with diver direct observations and video recordings, suggest that more elongated species such as the oar-ribbon fishes usually orientate their body vertically [17]. This characteristic head-up position involves vertically using all of their long fins through the water column, sometimes rapidly [64]. Indeed, their appearance as non-robust fishes has been confirmed through histological analysis revealing soft pale muscle tissues. When disturbed, these species can swim rapidly, but briefly. On the contrary, veliferids, and lampridids usually swim horizontally in the typical teleost way, confirming in this case, a different evolutionary story. These species are considered powerful swimmers, using their large pectoral fins for forward propulsion [48].

From the scarce data found in the literature, even if they are active predators, Lampriformes seem not to be aggressive fishes, and are provided with extraordinary features to offend prey or defend themselves from predators. The above-mentioned head-maxillae structure provides them efficacy in predation. At the same time, from a defensive point of view, fast swimming seems to be the primary mechanism of escape, with some exceptions. Particularly curious is the case of the regalecid *Agrostichthys parkeri* (Benham, 1904), which has been reported to give a mild electric shock if handled [65]. Moreover, some species of the genus *Lophotus* and *Radiicephalus* seem to have specialized organs that, as defense mechanisms, expel a dark, squid-like ink through the cloaca if disturbed [61,66]. *T. trachypterus* (Gmelin, 1789) showed a Batesian mimicry strategy when observed alive, and its feeding behavior seems to be strictly influenced by up-welling currents and the moon phases [17]. Moreover, the areal distribution of this species seems to be related to the movements of different aquatic masses within the water column [54]. Despite some cases of pathogenic infections that have been recorded in Lampriformes [57,67], no information in these species about the immune system and its role in organism defense is available [68,69,70].

Currently, no lampriform species are listed by the IUCN; however, it depends on the rarity of these fishes, and the scarcity of data about them. Considering the discussed role of Lampriformes in deep-sea food webs, fishermen and researchers should take more care in reporting the related data of their occurrence as by-catch [5,71], in order to deepen their stock evaluation, and consequently better assess if conservation measures are required.

## 3. Essential Systematics and Phylogeny of the Group

The taxonomy and systematics of Lampriformes passed through many insights in recent years, due to the advent of new molecular approaches to determining phylogenetic relationships [16,72,73,74]. For this reason, clarifying the taxonomy of the group is essential, to better evaluate and compare the literature in the field. Currently, the order Lampriformes comprises six families (Table 1): Lampridae (Gill, 1862), Lophotidae (Bonaparte, 1845), Radiicephalidae (Osorio, 1917), Regalecidae (Gill, 1884), Trachipteridae (Swainson, 1839), and Veliferidae (Bleeker, 1859) [75,76]. The family Lampridae consists of the genus *Lampris* (Retzius, 1799), which contains five species, three of which are very recent discoveries: *Lampris australensis* (Underkoffler, Luers, Hyde and Craig, 2018), *Lampris guttatus* (Brünnich, 1788), *Lampris immaculatus* (Gilchrist, 1904), *Lampris incognitus* (Underkoffler, Luers, Hyde and Craig, 2018), and *Lampris megalopsis* (Underkoffler, Luers, Hyde and Craig, 2018) [48]. The family Lophotidae contains two genera, *Eumecichthys* (Regan, 1907), which includes a single species, *Eumecichthys fiski* (Günther, 1890), and the genus *Lophotus* (Giorna, 1809), which constitutes four species: *Lophotus capellei* (Temminck and Schlegel, 1845), *Lophotus guntheri* (Johnston, 1883), *Lophotus lacepede* (Giorna, 1809), and *Lophotus machadoi* (Miranda Ribeiro, 1927) [76,77]. The family Radiicephalidae is constituted by only one genus, *Radiicephalus* (Osório, 1917), which contains two species: *Radiicephalus elongatus* (Osório, 1917) and the recently annotated *Radiicephalus kessinger* (Koeda and Ho, 2018) [78]. The family Regalecidae passed through many rearrangements that, over time, added or deleted species and genera that were considered synonyms or sisters. Currently, the accepted classification attributes two genera to this family, *Agrostichthys* (Phillipps, 1924), with *Agrostichthys parkeri* (Benham, 1904) as unique species, and *Regalecus* (Ascanius, 1772), which contains the two species *Regalecus glesne* (Ascanius, 1772) and *Regalecus russellii* (Cuvier, 1816). These species have several synonyms (e.g., *Regalecus kinoi, Regalecus masterii, Regalecus woodjonesi, Gymnetrus hawkenii, Gymnetrus russellii*) [75,76]. Similarly, the family Trachipteridae consists of three genera, *Desmodema* (Walters and Fitch, 1960), which contains the two species, *Desmodema lorum* (Rosenblatt and Butler, 1977) and *Desmodema polystictum* (Ogilby, 1898), the genus *Zu* (Walters & Fitch, 1960) with two species, *Zu cristatus* (Bonelli, 1819) and *Zu elongatus* (Heemstra and Kannemeyer, 1984), and the most prominent genus, *Trachipterus* (Goüan, 1770), with its six species: *Trachipterus altivelis* (Kner, 1859), *Trachipterus arcticus* (Brünnich, 1788), *Trachipterus fukuzakii* (Fitch, 1964), *Trachipterus ishikawae* (Jordan and Snyder, 1901), *Trachipterus jacksonensis* (Ramsay, 1881), and *Trachipterus trachypterus* (Gmelin, 1789). Several species of the Trachipteridae family have synonyms (e.g., *Trachipterus misakiensis, Trachypterus altivelis, Trachypterus nigrifrons*) [75,76]. The family Veliferidae is constituted by the genus *Metavelifer* (Walters, 1960), with the species *Metavelifer multiradiatus* (Regan, 1907), and the genus *Velifer* (Temminck and Schlegel, 1850), which contains *Velifer hypselopterus* (Bleeker, 1879) as its unique species [75].

The older classification also comprised the family Stylephoridae, which was recently moved on the basis of molecular data into the new separate order of Stilephoriformes, which contains one monotypic genus, *Stylephorus* (Shaw, 1791) [15,79,80]. The current review manuscript represents one of the first documents based on this new classification.

Different phylogenetic surveys conducted with morphological and molecular approaches have placed the Lampriformes order within the Acanthomorpha clade [81,82]. Lampriform fishes are primitive compared to the Percomorpha, but their precise placement among basal Acanthomorpha remains undetermined [51]. The monophyly of Lampridiformes is based on four apomorphies, three of which are correlated, and involve evolutionary modifications of the unique feeding mechanism. In these species, the maxilla slides forward with the premaxilla during jaw protrusion [61]. The Veliferidae family is deemed the sister group of all other lampriforms, with the oarfish and related that evolved from a common velifer-like ancestor during the late Cretaceous or early Eocene [1]. Effectively, the order Lampriformes is considered one of the ancient sister taxa to approximately 60% of all known teleost species [51,61]. This pivotal systematic position makes the order essential to evolutionary researchers, considering the difficulty of establishing this kind of phyletic relationship in classifying Acanthomorpha fishes [83].

In this regard, the inclusion of fossils in the analysis also assumes importance in phylogenetic studies since they deepen the knowledge of morphological characters and their evolution. During archaeological research in the Mediterranean area, some skeletal evidence of about another ten extinct Acanthomorpha species have been found in fossil findings [14,84,85]. These fossil relics have been related to ancestral Lampriformes [14,81,85,86]. In 1999, Sorbini and Sorbini described a fossil of the oldest known lampriform fish, *Nardovelifer altipinnis*, found in the Cretaceous deposits of Nardò, Italy [62]. Carnevale and Bannikov [87], and Papazzone et al. [88] described in Eocene deposits from Verona, Italy, two ancient species, *Bajaichthys elegans* and *Veronavelifer sorbinii*. Carnevale [84], reported about the first fossil of the ribbonfish *Trachipterus mauritanicus* from a Miocene locality in northwestern Algeria. Additional fossil taxa from other areas of the world reported the existence of some other Lampris-like species that were discovered in Miocene deposits in California (*Lampris zatima*) [89], two Oligocene lophotids, *Protomecicthys* and *Protolophotus* [85], and in Oligocene deposits from New Zealand (*Megalampris keyesi*) [90].

### Lampriformes Phylogenetic Relationships Based on mt-COI Sequences

The taxonomy and systematics of the order Lampriformes are still affected by the absence of a wide database, especially from a molecular point of view, which currently represent the main goal of this research [64]. Particularly for the rarest families, the few or incomplete descriptions joined to the morphological variation within, and similarities between Lampriformes species, lead to some uncertainty on phylogenetic relationships [91]. Molecular analysis will be essential to better assess the taxonomic status of these taxa, which will probably pass-through revisions in the coming years.

**Figure 1:**
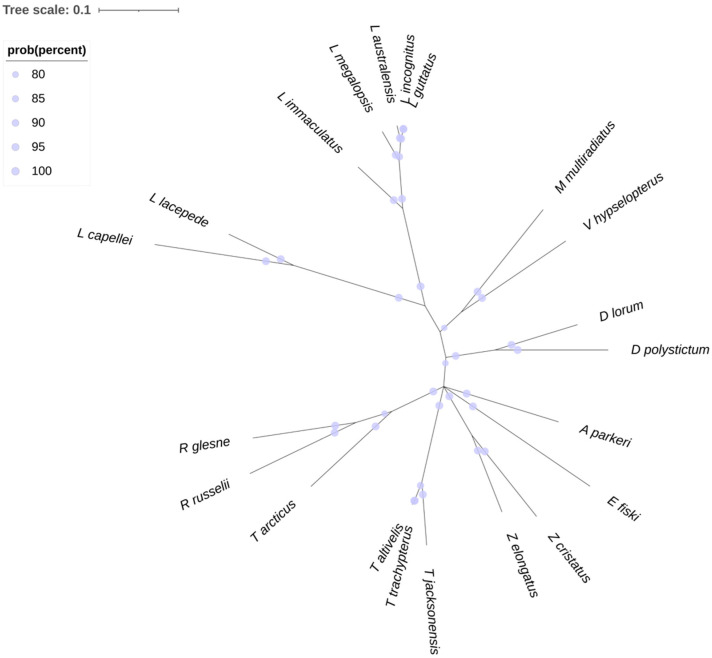
Unrooted radiation tree illustrating the phylogenetic relationships of the Lampriformes species based on the currently available mt-COI sequences.

Here, we report the results of the phylogenetic reconstruction of Lampriformes based on the currently available cytochrome oxidase subunit 1 (mt-COI) gene sequences revealed, with high posterior probability support that the topology of the tree is partially in accordance with the currently accepted taxonomic relationships amongst the families (Figure 1). All the coding sequences of mt-COI from 21 Lampriformes species, currently annotated in the GenBank database (Supplementary Materials Table S1), were aligned with MUSCLE and then trimmed with Gblocks. A total of 542 positions were selected for Bayesian phylogenetic reconstruction using the GTR+I nucleotide substitution model. The consensus tree was built after burning 25% of the trees from 500,000 generations. Bayesian posterior probabilities are represented as percentages. This analysis was performed with the pipeline NGPhylogeny.fr [92]. Despite having chosen the most common gene used for phylogenetic analysis in teleosts, there remained six species included in the order (*L. guntheri, L. machadoi, R. elongatus, R. kessinger, T. fukuzakii, T. ishikawae*) that were without annotated mt-COI sequences, denoting the strong lack of data on the subject, and the need to deepen research in this topic.

Particularly, no available data were present for the entire family of Radiicephalidae. On the contrary, all the species of the Lampridae, Veliferidae, and Regalecidae families had mt-COI sequences available on NCBI. From our analysis, the members of the first two taxa clustered in accordance with the current taxonomy, on two monophyletic branches that are not distant from each other. On the other hand, the three species of the Regalecidae family were grouped in two separate branches from the same node, one related to the two species of the genus Regalecus, while the other one consisted only of *A. parkeri*. Beginning from the same node, there originated three other branches, two of which related to the Trachipteridae genera, *Trachipterus* and *Zu*, grouped as expected, and the other one concerning the species *E. fiski* was separate from the other Lophotidae. Indeed, the species of the genus *Lophotus* grouped somewhat distantly from *E. fiski,* in a branch near the Lampridae, as a well-defined clade. This represents the most important difference between our analysis and the accepted phylogeny of the order. Moreover, the two species of the genus *Desmodema* clustered separately from the rest of the Trachipteridae genera, without being too evolutionarily distant. It is also interesting to note how *T. arcticus* grouped separately from its congeners, on a shared clade with *Regalecus* species, which originated from the same node as the other *Trachipterus*. We can find the most interesting insight from our analysis of the position within the Lophotidae and Trachipteridae families. Further and more complex analyses (e.g., phylogenomics) are necessary, in order to better assess these relationships, especially those that are between quite dissimilar organisms, both morphologically and biologically, such as Lophotidae [85].

## 4. Distribution of Lampriformes Orders in the Mediterranean Sea

Specimens of Lampriformes order are rarely seen alive, especially in the adult stage. Very few cases were recorded and shared online regarding shabby animals in coastal areas or watered on the surface [4]. Some expert scuba diving photographers have caught juvenile forms of some lampriforms on camera, showing the incredible morphology that characterizes most of the group in this life stage [2]. Occasionally, relatively small specimens have been taken during scientific surveys using midwater or bottom trawls [24,44,93,94,95]. Among the Lampriformes, Lampridae and Veliferidae comprise species that inhabit shallow coastal waters, mainly distributed in the Indian and Pacific Oceans [61]. Sometimes, specimens of Trachipteridae and Regalecidae can be found in surface waters, but more by chance than by choice, driven by vertical currents [96]. Based on the literature, the remaining lampriform families consist of mostly mesopelagic but often epipelagic and bathypelagic species, reported at depths beyond 2000 m [24]. This high depth range of distribution is common in eurybathic fishes, which, like Lampriformes, rarely show the typical decompression signs after the capture from depth bottoms [97]. The general distribution of the order covers all of the oceans worldwide [1,2]. Almost all these species are widely distributed in tropical and temperate zones, but they are considered extremely rare despite this typical distribution. From records in the literature, these species do not seem to inhabit extreme latitudes, apart from two species. One fish of the family Lampridae, the rare *L. immaculatus*, was reported in the sub-Antarctic zone (Falkland–Patagonia) around 56° south latitude, in 1985 [98]. At the other pole, the trachipterids, *T. articus,* can inhabit as far as the 60th parallel north [99].

As the main aim of this manuscript, an extensive literature review on the records of the Lampriformes families in the Mediterranean Sea is hereafter individually treated.

### 4.1. Lampridae (Gill, 1862)

The family Lampridae, commonly named opahs, is currently represented by the single genus *Lampris* that is characterized by a compressed oval-shaped body, covered with very smooth and small scales, brightly colored with a dorsally bluish-sighted bottom that becomes paler reddish ventrally; it has very visible fins tending to bright red, with the entire body exhibiting scattered round silvery spots [1,48,100]. The dorsal and anal fins are elongated and are retractable in a dedicated slot to facilitate swimming. Indeed, they are the most skilled group in swimming within the Lampriformes order [101,102]. The maximum size reported is around 190 cm in length and over 250 kg in weight, making it the heaviest representative of the order [103]; for shape and size, opah is often confused with the Atlantic sunfish *Mola mola*. The species of this family have coastal habits, and appear to prefer shallow and warm waters, but occasionally they have been found at greater depths (up to 500 m) off the coasts [101,104]. Lamprids possess a small toothless mouth through which they prey on small cephalopods, fishes, and crustaceans [105,106]. However, it is an opportunistic family, and some authors have also reported the occurrence in their stomachs of clams and crabs. Occasionally caught in longline fisheries intended for large pelagic such as tunas and swordfishes, lamprids have the most appreciated flesh among the order Lampriformes, and it is marketed as by-catch [98,107]. Due to its coastal nature, the species *L. guttatus* is often caught by amateur fishermen with various fishing methods, and is therefore, one of the rare Lampriformes species that is occasionally fished in some areas, even from the shore.

During an interesting study, Runcie et al. [104] investigated the capacity of *L. guttatus* to maintain a cranial endothermy. From their results, the authors concluded that this capacity was developed in opah through the proximal region of the paired lateral rectus extraocular muscle, which works in this species as a source of heat. The higher citrate synthase activity of this muscle highlights the higher capacity for aerobic heat production. Moreover, in *L. guttatus*, this muscle is insulated by a layer of fat, and is surrounded by a network of arteries and veins which functions as a heat exchanger. This endothermal capacity of *L. guttatus* was also studied by Wegner et al. [108], who investigated the whole body endothermy of this species, relating it to a constant movement of pectoral fins, and to the particular structure of the gills, which are equipped with heat exchanger structures. Bo and colleagues [109] reported in this sense some recent insights on *L. megalopsis.* The investigation was conducted from a genetic point of view, following the whole genome sequencing of the species, which led to the detection of positive selection on several genes that were known to be involved in muscle differentiation and development. This feature could represent an important key for the opahs to expand their distribution habitat, both in a geographical and bathymetrical way, exploiting their thermal tolerance to move into deeper and colder waters. Moreover, it represents a very rare feature among fish species, worthy of future in-depth analysis.

**Figure 2:**
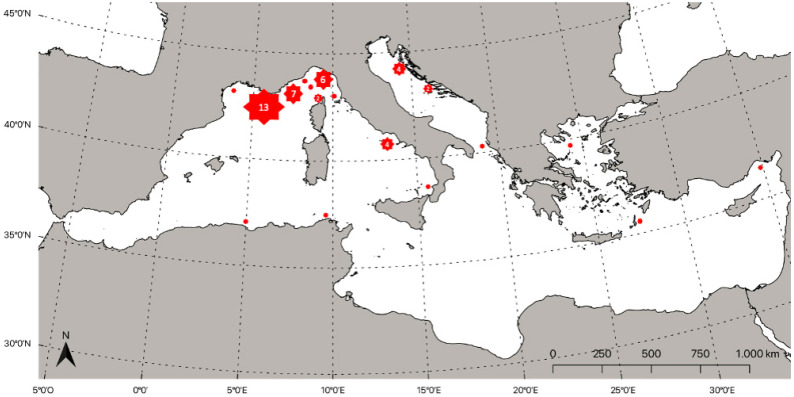
Distribution map of the Lampridae family occurrence in the Mediterranean Sea, in this case, of the unique species reported in the basin, Lampris guttatus. Red stars show the occurrence of a single specimen; the number inside a star refers to the occurrence of the specified number of specimens in the area.

Distributed worldwide in tropical and temperate waters, mainly reported in the North Pacific Ocean [48], and in the Mediterranean Sea, the family Lampridae is represented only by the species *L. guttatus*, which was anciently reported in the Ligurian Sea by Spinola in 1807 [110], and Risso in 1826 [111] (Table 2). Its occurrence in the North-Wester area of the basin was confirmed by several other authors [112,113,114,115,116]. The presence of this species was successively detected in some other areas of the Mediterranean basin, with an increase in distribution in the last 50 years. In the central part of the basin, the presence of *L. guttatus* was detected in the southern Tyrrhenian Sea [117,118], and in both the Italian and Albanian parts of the Adriatic basin [119,120,121]. Particularly for the Adriatic Sea, the presence of the species was reported as *Lampris luna* by Katurić in 1902 [122], and successfully by Cnrković in 1957 [123]. Moving to the eastern Mediterranean Sea, the species was previously detected in the Aegean Greek Sea by Sinis [124]; successively, Corsini-Foka [125] reported the occurrence of a specimen captured by longline fisheries near the surface, off Pigadi in Karpathos Island, the central part of the Aegean Sea. More recently, the species was detected in some new areas, such as in the eastern Mediterranean in Turkey [126], and in the central-eastern part of the basin in Tunis [127]. Francour et al. [128] reported, in 2010, that at least 23 specimens in the western Mediterranean along the French coasts were collected between 1997 and 2009. In the same study, the occurrence of the opah was reported in Gouraya, in northern Algeria. Considering the massive presence of *L. guttatus* in the area between the Gulf of Lion and the Ligurian Sea, it is probable that the northwestern Mediterranean represents the species’ preferred area in the basin. The estimated distribution of records is shown in Figure 2.

### 4.2. Lophotidae (Bonaparte, 1845)

The family Lophotidae, commonly named crestfishes, consists of two mesopelagic fish genera, *Eumecichthys* and *Lophotus*; the first is monospecific, while the second comprises four different species [53]. The elongated and compressed morphology of the body, which sometimes can reach 2 m in length, is characterized by a large crest or horn that extends the jaw in the species of genus *Lophotus*, and protrudes far forward of the jaw in genus *Eumecichthys* [1,50]. The scales are absent, except for the lateral line ones that show the characteristics of tubular morphology, which are shared between some Lampriformes families. The color of the body is silver, with numerous dark vertical bands in *Eumecichthys*; in contrast, in *Lophotus*, the bands are absent, and the body color tends to be blue dorsally, grading to silver ventrally, with multiple silver/white spots. As with most Lampriformes, the long evident dorsal fin is reddish; the pectoral, pelvic, and caudal fins in some species are absent (especially pelvic fins) or reduced (caudal fin). The upper jaw is protrusible-armed on the jaw and vomer, and has small conical teeth that are used to prey on cephalopods and small fishes [129]. Crestfishes can use a tubular gland located in the back intestine that can emit through the anus an ink-like black liquid to deter predators [66,130]. No fisheries information has been reported on these fish, which are considered rare and free of commercial interest. Moreover, the group’s taxonomy is uncertain, especially for the genus *Lophotus*, whose main species seems to be *L. lacepede*; meanwhile, other species require further molecular investigation to be better clarified [83,120].

From the scarce data in the literature, the distribution of crestfishes seems to comprise all of the oceans at warm latitudes, recorded from the surface to about 1000 m of depth [131]. Two specimens of *L. guntheri* were recently reported for the first time in Taiwan by Koeda and Ho, in 2017 [130]. Despite this, several other authors historically reported the presence of this family in Asian waters [132,133]. Moreover, Craig and colleagues reported in 2004 the presence of this family in all areas of the Pacific Ocean, and in part of the Atlantic [134].

The Mediterranean distribution of the family Lophotidae is affected by a data deficiency due to the rarity of these fishes, but probably to a lower presence in the basin in comparison to other families of the order [131]. In the Mediterranean Sea, the presence of a single species of Lophotidae, *L. lacepede*, was reported, having been occasionally caught with longline or trawling fisheries (Table 3). The occurrence was originally reported in 1890 by Kolombatović, followed by other reports, all from the Adriatic Sea before 1950 [135,136,137]; some other authors followed with reports of the presence of the species in the central Mediterranean Sea [116,138,139,140]. Within the Adriatic sea, the presence of *L. lacepede* was confirmed at least six times by other authors [120,141,142].

**Figure 3:**
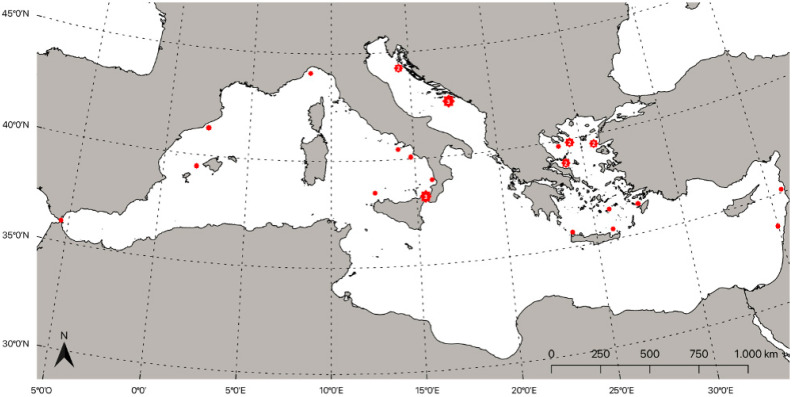
Distribution map of the Lophotidae family occurrence in the Mediterranean Sea, in this case, of the unique species reported in the basin, Lophotus lacepede. Red stars show the occurrence of a single specimen; the number inside a star refers to the occurrence of the specified number of specimens in the area.

Regarding the central part of the basin, Tortonese reported, in 1970, the occurrence of *L. lacepede* in the Strait of Messina [116], without providing specific details. However, one year later, Magazzù and Zaccone reported the same occurrence in this important ecological area [143]; another record confirmed the presence of this species in 1980 [144]. Several authors also reported the occurrence in southern Tyrrhenian waters [145,146,147]. In 2017, Falsone et al. reported the occurrence off San Vito Lo Capo (northeastern Sicily, Italy) coast of a *L. lacepede* specimen caught with a longline drifting fishery, a typical system used in this area to catch common dolphinfish (*Coryphaena hippurus*, Linnaeus, 1758) [3]. In 1979, a specimen was reported from the western Mediterranean Sea by Portan and Del Cerro [148]. After one year, in 1980, Rey reported the capture of one specimen in the Strait of Gibraltar area [149]. Its presence in the western part of the basin was confirmed in 2005 by Rodriguez et al., with the report of *L. lacepede* in a larval stage in the Balearic region [150]. The eastern part of the Mediterranean Sea seems to be the preferred area for this species in the basin, which was also reported previously by some other authors. The first documented record in the southern part of this area was by Bilecenoglu et al. in 2001, from Gökova Bay [151]. An interesting report of an adult specimen that was 650 mm in length occurred at 329 m, in 2017, in the northern part of the Turkish Aegean Sea [152]. Minos et al. and Minasidis and Kaminas reported respectively, in 2015 and 2021, of the occurrence of *L. lacepede* from the northern part of the Aegean Sea in Greece. In 2019, Aga-Spyridopoulou et al. reported, through a citizen science project, the occurrence in the central and northern Aegean Sea of five specimens, detected between 2016 and 2018 [153]. In the same year, Yapici reported an occurrence in the eastern part of the Mediterranean basin of a specimen that was photographed by a scuba diver, and successively identified as *L. lacepede* in the Levantine Sea (Turkey) [154]. A very recent manuscript reported, for the first time, an occurrence of the species off the coast of Syria [155]. Figure 3 shows the estimated distribution of the mentioned records.

### 4.3. Radiicephalidae (Osorio, 1917)

The family Radiicephalidae comprises the genus *Radiicephalus*, with two species currently annotated; one of these, *R. kessinger*, was very recently described as a new holotype by Koeda and Ho [78]; based on a molecular approach, it was separated from the historical one, *R. elongatus*, originally described by Osório in 1917 [156]. Commonly named tapertails, compared to the previously described families, these fishes are characterized by smaller dimensions of the body that retain the typical morphological features of the order [1]. Indeed, they present an elongated and compressed naked body, silver-colored with evident red fins. The dorsal fin inserts over the eye and develops along the entire body length [157]. The long caudal fin forms a posterior projection that, in live specimens, can reach body length. Unfortunately, as for all of the Lampriformes, it is difficult to find undamaged specimens that can perfectly show these peculiar features [158]. The anal fin is reduced and distinguishes tapertails from the members of the Trachipteridae family in which it is absent. Scales are absent, except for the lateral line ones that are tubular. A distinctive characteristic of this family is the fourth, fifth, and sixth preural center, with elongated haemal spines that pierce the ventral margin of the body [1]. Similarly to Lophotidae, they possess a defense mechanism that is based on a similar ink gland [50,66] which is used when threatened. As for the other members of the order, radiicephalids show the protrusibility of the upper jaw, which is toothless and is used to prey on small fishes, crustaceans such as euphausiids, and molluscs in meso- and bathypelagic environments [157]. The species of this family are considered very rare, and few records have occurred until now; hence, the knowledge of this group is the poorest of the entire order, and further records are necessary.

The confirmed distribution of this family spans the Atlantic Ocean, from Spain to South Africa, and the eastern Pacific [138,157,158]. A recent new annotation of *R. kessinger* in the western Pacific Ocean (Taiwan) represents the first confirmed report of the Radiicephalidae family in this geographical area [78,130]. From the original description of Osòrio from the Atlantic coastal zone of Morocco [156], the presence of the species in this area was successfully confirmed by some other authors, also by juvenile specimens that are always important to establish nursery areas [159]. Some other records occurred in the eastern Atlantic area to the southern part of South Africa [91,157,160,161,162]. Similarly, the eastern Pacific Ocean was confirmed to be a suitable area for the species by some authors [163,164,165].

There is no record of *Radiicephalus* specimens in the entire Mediterranean Sea, suggesting the absence of these species in the basin. However, their occurrence in the Atlantic area outside Gibraltar Strait highlights their preference for oceanic ecological and trophic conditions, and there is a possibility that these fishes could be present, but very rarely, in the nearby Mediterranean basin. Moreover, the morphology of this family reminds inexperienced eyes of the Trachipteridae family members that are most common In the Mediterranean Sea. For this reason, incorrect identifications of tapertails such as trachipterids may have occurred over time. It is also considered that these lesser-known and much smaller Lampriformes are not interesting from a commercial point of view and are rarely collected on scientific surveys. Specific studies in this regard are needed; however, because of the current literature, we confirm the absence of this family from the Mediterranean area.

### 4.4. Regalecidae (Gill, 1884)

The family Regalecidae is one of the most common and, consequently, is a well-known taxon of the order [166]. Currently, for this family, two genera are accepted, *Agrostichthys* and *Regalecus*. Commonly named oarfishes, to this group belong iconic species such as *R. glesne*, a fabled silvery fish of significant length (its maximum recorded length of about 17 m represents the longest of all bony fishes). It has bright crimson reddish fins, with long rays in the dorsal fin [1,2]. Some ancient sea legends and myths have been historically linked to this species, due to its morphology and size, and its habit of standing at the surface or swimming out of the water to stand on beaches, especially after windstorms, leading to its association with sea “monsters” [167]. The morphology of the entire family is characterized by a very elongated and compressed shape of the naked (except for tubular lateral-line scales) body, with a long dorsal fin consisting of over 400 elements in some species, with the characteristics of its first 8–10 rays being extremely flexible and elongated. Even the pelvic fins’ soft rays are significantly developed in length, while the anal fin is often absent or constituted by some few elongated rays. In regalecids, the dorsal and pelvic fin rays are provided with small spinules that project laterally, such as the caudal-fin rays, if present. The anal fin is absent, a feature that is shared with the Trachipteridae family [1,2].

A particular feature of this group is represented by its self-amputation (autotomy) ability [167]. This process involves only the posterior part of the body over the anus and the caudal fin. Self-amputation probably occurs more times during life because it does not involve any vital organ. The records on lengths over 1.5 m often show clear signs attributable to autotomy, such as a healed-over stump or “terminus”, with the lost part never regenerated. This mechanism may represents a defense strategy against predators, such as that found in many other examples in both marine and terrestrial organisms [167]. From the literature information, some insights on reproduction are known for this family. Sexually mature specimens meet and spawn, as broadcast spawners, between July and December in the North Atlantic Ocean, Mediterranean Sea (Straits of Messina), and in the South Pacific (New Zealand-Australia) [139,167,168]. Recently, Oka and colleagues [169] successfully performed artificial insemination, beginning with the gametes of two dead sexually mature specimens of *R. russellii* that washed up off the coast of Okinawa Island. This study obtained meaningful information on this species’ hatching and larval stages, such as the 18 days needed for hatching, as well as for morphological and behavioral description of the larvae.

The head structure is the same for most Lampriformes, characterized by a protrusible jaw that is used to prey on small fishes, cephalopods, and euphausiids [61]. Regalecids species have mainly mesopelagic habits, and are considered inedible; for this reason, even if sometimes they appear as by-catch in long-line or trawl fisheries aimed at pelagic species, they have no commercial value [50,71]. Despite this, due to its fascinating morphology, oarfishes are considered one of the most common Lampriformes groups (despite being a rare species); moreover, with the increase in the importance of citizen science, even the personal records of living oarfish are essential to acquire, to increase our knowledge on these species. Underwater footage has highlighted their ability to swim in a vertical position, commonly called head-up swimming, without muscular effort, but by exploiting the movements of their fins and long appendages. Probably, this habit is due to the feeding behavior of these species [167]. Recently, the complete mitochondrial genome of *R. glesne* was annotated by Yu et al. [170]; this represents an important step in deepening the knowledge of this species and its phylogenetic position.

The Regalecidae family is distributed in all the oceans, even at high latitudes, from the surface to about 1000 m deep. However, only *R. glesne* seems to have a confirmed, worldwide distribution [4,139,171]. In 2002, Roberts reported about a specimen of *R. glesne* that some years before beached at a naval war base in southern California [172], and reported exciting information on some myths that have been linked to this fish. Other authors also confirmed the presence of oarfishes in the Atlantic Ocean in several areas [139,166,167]. However, the Pacific Ocean represents the geographical area with the most significant number of records in the literature. Both species of genus *Regalecus* and the one of genus *Agrostichthys* occurred in their various zones [173,174,175,176]. While the genus *Regalecus* is widely distributed in all the oceans, the genus *Agrostichthys* occurs only in the southern oceans and appears to prefer these latitudinal areas [2].

Regarding the distribution of the family Regalecidae in the Mediterranean Sea, only the genus Regalecus was found, in 1826, when Risso reported the occurrence of two regalecids in Nice under the name *Gymnetrius gladius* [111]. Many other authors also reported the occurrence of these species in subsequent years, using different specific names until the current annotation was made by Gill in 1884. The distribution of these ancient reports is strictly connected to the central part of the Mediterranean basin, comprising the geographical zone between the Ligurian Sea and the Gulf of Lion area [114,177,178,179,180]. Some more recent authors confirmed the presence in this area of Regalecus [116,145,181,182,183]. Lozano-Cabo reported, in 1969, the occurrence of an oarfish specimen from Mazzarrón, in the Spanish western Mediterranean waters, highlighting its very rare presence in this area of the basin [184]. Indeed, this remains a unique record from the western Mediterranean.

Some ancient reports regard the central part of the Tyrrhenian Sea [113,180]; an occurrence was recently confirmed by Psomadakis et al. in 2012, with a record of *R. glesne* from Terracina [145]. The presence of this species was also confirmed in the southern Tyrrhenian sea by some authors [185,186] that reported its occurrence in the area near the Strait of Messina from the other Lampriformes occurrence data, this seems to be an exciting area to study the entire order in more depth. Another geographical area where the presence of *R. glesne* was recorded was in the Adriatic Sea, from both the Italian and Croatian coasts. The occurrence of this species was reported as *Regalecus gladius,* in 1933 by Padovani, in the northern part of this sub-basin [187]; more recently, two different authors confirmed this occurrence (with the currently accepted name) in the central Adriatic sea [8,188]. Dragičević recorded at Palagruža Island the occurrence of the early life stage of the species, an essential reference to deepen the study of breeding areas in the Mediterranean basin.

**Figure 4:**
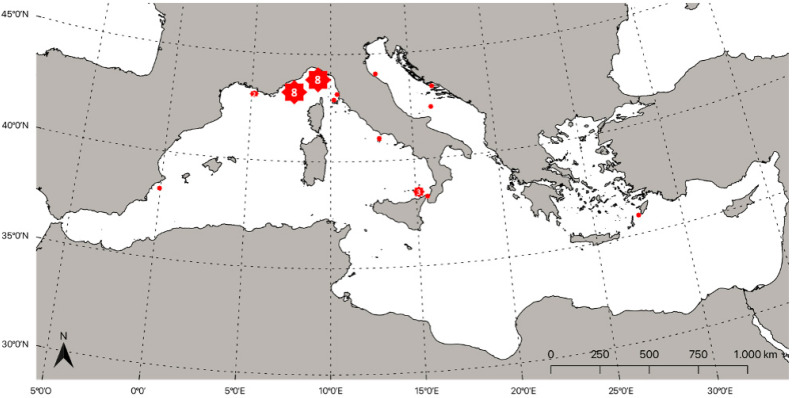
Distribution map of the Regalecidae family occurrence in the Mediterranean Sea, in this case, of the unique species reported in the basin, Regalecus glesne. Red stars show the occurrence of a single specimen; the number inside a star refers to the occurrence of the specified number of specimens in the area.

Only a few authors have recently reported the presence of *R. glesne* in the Aegean Sea [189,190], which, however, was confirmed by Corsini-Foka in 2009 [125], who reported the occurrence of one specimen in 1993 off the coast of Gennadi (Rhodes). From the review of the reports that are currently available in the literature regarding the Regalecidae family, only the presence of *R. glesne* was confirmed in the Mediterranean Sea (Table 4). In detail, the northern part of the central Mediterranean Sea seems to be the area of preference for this species (Figure 4). However, ancient records present some problems related to the different nomenclature of the species over the past centuries. The ancient morphological identification of these fishes is not entirely reliable and should be considered with caution. Moreover, it cannot be neglected that some of the records that were reported in the list were not caught fish, but were observed alive; thus, it was probably not simple to discriminate between the two *Regalecus* species, in our opinion. Nevertheless, these fishes are considered rare in the Mediterranean basin.

### 4.5. Trachipteridae (Swainson, 1839)

The family Trachipteridae comprises the common-named dealfish and ribbonfish; it is one of the most widely distributed taxa of the Lampriformes order. Currently, three genera are accepted: *Desmodema*, *Trachipterus*, and *Zu*, which include ten different species [61]. The largest one is Trachipterus, which comprises six species of dealfish. An elongate and compressed ribbon-like body characterizes the entire family. Notably, in genera *Desmodema* and *Zu*, the body depth decreased reaching the caudal peduncle; this shape development resulted more accentuated in *Zu* [1]. The bright, silvery body sometimes shows dark spots (*Desmodema* and *Zu*) or vertical oblique bars (*Trachipterus*), and is adorned with crimson reddish fins, which are characteristics of the entire order. The dorsal and pelvic fins are usually highly elongated, more so in larval stages than in adult specimens, in comparison to body length. Furthermore, the caudal fin shows the upper lobe rays to be widely elongated as a palette, especially in *Zu* [2]. The anal fin is absent, and this feature helps researchers discriminate between this family and the Radiicephalidae, in which the anal fin is present, even if reduced.

Unlike most Lampriformes, a few small and deciduous scales are present in the dealfish of the genus *Trachipterus* [191]. The lateral line scales are tubular and present sharp spines, a characteristic that is sometimes used in identifying them [192]. Moreover, lateral spiny fin rays, which are found in oarfishes’ dorsal, pelvic, and caudal fins, are shown by trachipterids but in this case, are better developed. Regarding dimensions, the genus *Trachipterus* seems to reach 3 m in body length, even if detailed records in the literature, with the collection of morphometric and meristic data, mainly regard specimens with a maximum length of 1,6 m [116]. The maximum recorded length for *Zu* and *Desmodema* was about 1,4 and 1,1 m, respectively, confirming the smaller size of these genera compared to *Trachipterus* [64].

As a distinctive feature of the entire order, in trachipterids, the absence of an anterior palatomaxillary ligament and palatine prong leaves the axis maxilla-premaxilla free to extend during jaw protrusion [157]. Trachipterids use this buccal apparatus to feed on pelagic cephalopods, crustaceans, and small fishes. The significant quantity of data for this family permit better exploration of the feeding behavior of these fishes, which seem to be omnivorous, and also includes terrestrial food such as fragments of pollen cones of pine, petals of terrestrial plants, other fragments of terrestrial grass, and beetles [193]. They also feed on aquatic plants and algae, as well as on different taxa of crustaceans, such as Cladocera, Copepoda, Decapoda, and Isopoda [54]. They are meso-bathypelagic fishes that inhabit a wide distribution between surface (mainly for larval stages) and deep-sea environments (up to 2000 m) [24].

Very little is known about their reproductive biology, but the eggs are free-floating, large, and red, similar to those of other families of the order [6]. Interesting notes on larval/juvenile stage features come from a recent study in the Mediterranean Sea, in which some specimens were observed alive in shallow waters during the early nighttime [17]. These specimens showed the typical head-up position during swimming and avoided being carried away by the current using just dorsal fin movements to maintain their buoyancy. However, when alarmed, a fast-swimming movement that used the whole body was shown. Furthermore, feeding behavior characterized by extremely fast protrusions of their jaws to prey on planktonic organisms was confirmed. From the observed behaviors, the authors also confirmed the moon’s influence on this species’ occurrence within the planktotrophic web and hypothesized a Batesian mimicry strategy used by the species to escape from predators.

The fragility of these fishes results in unavoidable damage whenever they are captured in trawling nets, which is the most common way to capture these fishes as by-catch, along with long lines for large pelagic fishes [5,71]. This feature leads to severe difficulties in morphological identification and discrimination between the vast number of species, and the revision of the literature requires care. For example, differentiating *T. trachypterus* and *T. arcticus* is only reliably accomplished through vertebral counts, which differ by about ten (84–96 vs. 99–102, respectively) [194]. Molecular approaches could lead in future years to new rearrangements of the family taxonomy [16].

The general distribution of the family Trachipteridae includes all the oceans, circumglobally [64]. Indeed, their presence was historically reported throughout the entire Pacific Ocean [192,195,196], Atlantic Ocean [197,198,199], and Indian Ocean [200,201]. Considering that all the three trachipterids genera underwent some taxonomical rearrangements, in the literature it is possible to find many reports using different scientific names for the same species.

Within the Lampriformes order, the family Trachipteridae is better represented in the Mediterranean Sea, which Bonelli reported for the first time in 1920, with the description of the species *Trachipterus cristatus*, which today is known by the accepted name, *Z. cristatus*. Indeed, from the literature, two different genera are present in this basin, *Trachipterus* and *Zu*, with three species in total, *T. trachypterus, T. arcticus,* and *Z. cristatus*. The genus *Desmodema* was never recorded in the Mediterranean basin [202], with any one of the two species, originally described in 1898 by Ogilby, *D. polystictum*, and the “recently” described one by Rosenblatt and Butler in 1977, named *D. lorum* [203].

The genus *Trachipterus* is well distributed in the Mediterranean Sea and is represented by the species *T. trachypterus* and *T. arcticus*. Regarding the latter species, only a single record occurred in the Mediterranean Sea, from the coasts of Spain not too far from the Gibraltar Strait [166]. However, the presence of this species in the Atlantic Ocean could influence this occurrence; moreover, the record was not well documented. Regarding the Mediterranean dealfish *T. trachypterus*, apart from the documented record that was analyzed in this review, some authors reported on the relative common occurrence of this fish (even with several personal communications). Costa, in 1991, reported in his atlas about the usual occurrence in the Strait of Messina area of some specimens of Mediterranean dealfish [204].

Furthermore, some authors recorded and confirmed the occurrence of dealfish in the Aegean Sea [140,151]; despite this, the occurrence of *T. trachypterus* in this area remains rare. Jardas, in 1980, reported about the occurrence, in the previous hundred years, of at least forty-six adult specimens from the Adriatic Sea [205]. The Adriatic Sea represents one of the most prevalent areas in the Mediterranean basin for these species, with several records of various life stages of *T. trachypterus,* from 1881 to recent years [116,136,193,205,206,207]. Some of these records come from the Gulf of Trieste in the northern Adriatic Sea. It is interesting to note how sometimes the specimens were found to be in poor health conditions, as reported by Borme and Voltolina [54]. Another ecologically interesting feature linked to the occurrence of *T. trachypterus* in the Adriatic Sea is the inconsistency of its records [208]. Indeed, the presence of this species in the basin, especially in the northern part, seems to be related to oceanographical and climatological parameters that are linked to the input of intermediate waters in the basin, which influence the water characteristics [54]. These thermohaline anomalies coincide with the rare species found in the Adriatic Sea, Trachipterids included, as reported by Jardas and Pallaoro [209]. The occurrence of *T. trachypterus* in the Ligurian Sea, historically reported by Tortonese [210], was confirmed by Garibaldi in 2015 [5], with an interesting analysis of the local longline swordfish fisheries by-catch. The manuscript was based on the percentage of observed non-commercial specimens found on board, compared to the commercial ones, among which the species represented about 10% of the former. However, occurrence numbers were not reported.

The occurrence of the Mediterranean dealfish in the central Tyrrhenian Sea was initially reported by Cau in 1980 [211], with three specimens found off the Sardinia coast, and successively by Psomadakis et al. (2006) [118] and Tiralongo et al. (2020) [212], who recorded one specimen from Anzio and Pianosa, respectively. Recently, it was well assessed by Macali and colleagues in 2020, in a fascinating study from Ponza [17], which provided some significant findings on the species. During their study, the authors observed and recorded eighteen live specimens via scuba video equipment, of which four were collected and analyzed. This study provided essential information on this species’ biological and ecological features, such as the influence of upwelling currents on their presence in shallow waters, and the swimming style used in vertical positions, with some good video references. The presence of the species in the eastern Ionian Sea was recently recorded by Mytilineou and colleagues in 2010, with a record from Cephalonia Island [213]. This occurrence was confirmed by three more recent records from the western and central Ionian Sea, reported by Tiralongo et al. [24].

Regarding the eastern part of the Mediterranean basin, the presence of *T. trachypterus* was reported by Golani (1996) [214] from the Israelian Levantine Sea. More recently, Yapici reported on a juvenile specimen that was video recorded in 2016 from the Çeşme coastline, representing the first record from the Turkish Aegean Sea [154]. More recently, Gökoğlu and Özen (2021) recorded an occurrence of the species from the Gulf of Antalya, extending the information collected from the Turkish waters [215]. The occurrence in the Aegean Sea was recently confirmed by Kaminas et al. (2021), with a conference poster based on citizen science contributions, which reported six different specimens observed in Aegean waters [216].

The genus *Zu* constitutes two species, *Z. elongatus* and *Z. cristatus*, whose general distribution covers all the oceans, even if not in an equal way. Indeed, these fishes are more commonly recorded in some areas, such as the Pacific Ocean [78,91,138,201,217,218]. Moreover, the distribution of the two species is not comparable, especially in particular areas such as the Mediterranean Sea, in which many authors assessed the presence of *Z. cristatus*, while *Z. elongatus* was never recorded, and was considered absent [2,64,76]. In the western Mediterranean basin, the scalloped ribbonfish was originally reported in the Balearic Sea as *Trachiptenes cristatus* by Oliver in 1955 [219]. Ibáñez and Gállego (1974) reported about an occurrence from Blanes (Spain) [220], and successively other two specimens were reported in detail by Roig and Demestre, in 1982, from the same area on Cataluña [221]. More recently, García–Barcelona et al. (2014) reported on the occurrence in swordfish longline fisheries of two *Z. cristatus* specimens off the coasts of the Balearic Islands.

Moving to the central part of the Mediterranean Sea, many records of *Z. cristatus* occurred in the Ligurian Sea, beginning with the original description (with the original name of *T. cristatus*) of the species by Bonelli, who in 1920 reported on the capture in La Spezia of a 70-centimeter specimen, with crustacean and cephalopod remains in its stomach [222]. Several other authors successfully reported this species’ presence in this area [49,97,223]. Garibaldi (2015) recently included in his analysis of local longline swordfish fisheries by-catch, the presence of this species, over the already mentioned *T. trachypterus* [5]. In this case, the percentage of scalloped ribbonfish that was observed on board among the non-commercial specimens, compared to the Mediterranean dealfish percentage, more than doubled, representing about 25% of the non-commercial species. Unfortunately, the raw data were not reported, but it is conceivable that over 50 specimens were observed during the study.

Some other reports regarding the occurrence of the species came from the central/south part of the Tyrrhenian Sea [118,211,224]. Falsone and colleagues recently reported a specimen that occurred off the coast of San Vito lo Capo (Sicily), along with some important information about age and sexual maturation [3]. Indeed, from the otolith and vertebrae analysis of the adult specimens that were about 88 cm in total length, eleven accretion rings were counted. Moreover, the gonads of the male specimen caught during the summer of 2015 were developed to maturity stage 3. In the same year, Zenetos et al. [44] reported the occurrence of *Z. cristatus* that was unusually captured with a fishing rod in Vibo Valentia (south Tyrrhenian Sea). Two recent studies by Tiralongo and colleagues collected some different occurrences in the central part of the basin involving all of the Tyrrhenian Sea in latitudinal extension (from Capraia to Vibo Valentia), and the Ionian Sea [24,212]. In 2012, Bradai and El Ouaer [225] studied a specimen in depth, which was captured in 1954 in Tunisian waters (Gulf of Tunis) previously described by Postel in 1955 [226], and was conserved in the oceanographic museum of the Institut National des Sciences et Technologies de la Mer (Salammbô, Tunisia). Comparing that specimen with a new one that occurred in 2009 in Mahdia, the authors confirmed the presence of *Z. cristatus* in the southern part of the central Mediterranean Sea [225,226].

Regarding the Adriatic Sea, in a similar way to *T. trachypterus*, Jardas reported in 1980 that at least 16 specimens of *Z. cristatus* occurred between 1946 and 1973 in the central and north Adriatic Sea [205]. In 2002, Dulčic reported the occurrence between planktonic samples of some eggs that were attributable to *Z. cristatus*, as well as some important information on their morphology and features [6]. Indeed, the author confirmed the autumnal occurrence of the eggs of this species in accordance with the previously hypothesized spawning period. The same author reported, along with some colleagues in 2014, the occurrence of three separate juvenile specimens from Croatian waters [227]. The occurrence in the Aegean Sea was recently confirmed by Kaminas et al. [216], with a poster presentation based on citizen science contributions, which reported two different specimens observed in the Dodecanese Islands and Levos Island, respectively.

**Figure 5:**
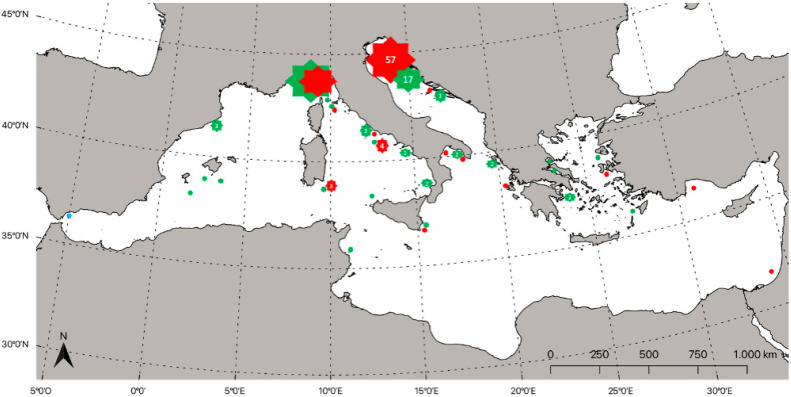
Distribution map of the Trachipteridae family occurrence in the Mediterranean Sea. The three species reported in the basin were identified by different colors of stars. Blue stars indicate the distribution of Trachipterus arcticus; red stars indicate the distribution of *T. arcticus*; green stars indicate the distribution of *Z. cristatus*. Small stars show the occurrence of a single specimen; the number inside a star refers to the occurrence of the specified number of specimens in the area; for the Ligurian Sea, the numbers are not indicated for the distribution of *T. trachypterus* and *Z. cristatus* because they were not shown in some studies; however, the sizes of the stars estimate the quantities.

Considering the above reported wide distribution in all areas of the Mediterranean basin (Table 5), and that this fish occurred in the same manner as all the other families (as by-catch in trawling nets or longline fisheries in mid-depth and deep waters), it is reasonable to state that this family is more common, and better adapted to the biological and trophic conditions of the Mediterranean Sea. For this reason, more information about Trachipterids’ ecological interactions is available, and their role in trophic webs is more known and defined [54,228,229]. For this reason, they are even more interesting from a research point of view. Figure 5 shows the estimated distribution of the mentioned records.

### 4.6. Veliferidae (Bleeker, 1859)

The Veliferidae, commonly named velifers, is a family that includes two monospecific genera, *Metavelifer* with the species *M. multiradiatus*, and *Velifer* with the species *V. hypselopterus* [75]. This group is one of the rarest among the Lampriformes families, and information on them is very scarce. The two species of this family are of small size, especially when compared to the sizes from other families of the order [230]. *M. multiradiatus* and *V. hypselopterus* can reach 28 cm and 40 cm maximum length, respectively. The body of veliferids is compressed and disc-shaped, the dorsal is silvery while whitish in the ventral part, with some vertical dark bars in the sailfin of velifer *V. hypselopterus*, and with brown spots in the spiny fin of velifer *M. multiradiatus* [231]. The morphology of these fishes is characterized by a cranial crest consisting of frontal bones, and by evident dorsal fins (which led to the family’s common name) that cover the body entirely from the dorsal raising to the caudal fin. The dorsal fin is colored by evident yellow bands in *V. hypselopterus*, and by brown spots in *M. multiradiatus.* The first dorsal fin ray is equal in length to body depth in *V. hypselopterus* [232]. The anal fin presents the same features as the dorsal fin but is reduced in dimension. The forked caudal fin shows the same coloration as the dorsal fin. A thick, scaly sheath of skin lies at the base of portions of both dorsal and anal fins [230]. The external morphology is characteristic, and not mistakable with other Lampriformes; however, a particular characteristic of this group is the reduced number of vertebrae (33–46) compared to the more elongated species that can have over 150 vertebrae in some cases [1].

Veliferids are classified as demersal epi- and mesopelagic species, particularly *M. multiradiatus,* which can reach greater depths (maximum reported 240 m), while *V. hypselopterus* prefers shallower water [61]. No information about the trophic behavior of this family has been reported; considering the typical jaw’s morphology and body dimension, they probably feed on tiny pelagic organisms, such as other Lampriformes at the juvenile stages. The Veliferidae family is considered to be the sister group of all other Lampriformes [61]. For this reason, despite being very rare, it remains one of the most studied families from the point of view of phylogenetic relationships [85,86,87].

The distribution of these species appears to be very strictly reduced to the Indo-West-Pacific area of the world. Indeed, based on the records present in the literature, the presence of veliferids was reported in the waters of Mozambique and Madagascar, the Australian continent, India, Japan, Indonesia, and Hawaii [233,234,235,236]. Recently, five specimens of *V. hypselopterus* were found in trawling nets off the coast of Oman [237]. From the same area, Al-Mamry and Jawad reported in 2021 the occurrence of ten specimens (six females, four males) of sailfin velifer [232], along with the essential collection of biometric and meristic data that were very useful in deepening the knowledge of this rare species.

The occurrence of the Veliferidae family in Mediterranean Sea waters has never been recorded. As for the Radiicephalidae family, based on the current literature, we can state that this family is not present in the Mediterranean basin.

## 5. Final Considerations

Monitoring the distribution of living organisms is essential, especially in a global climatically changing period that exerts environmental pressure on all organisms. In response to this, living organisms modify their living range, both in geographical terms and, especially for aquatic organisms, through bathymetric movements. This topic is even more critical when it involves rare organisms, due to their specific characteristics, as in the case of the members of the order Lampriformes. The literature review showed that these organisms are within marine trophic webs along most of the water column, inhabiting it from the surface down to the abyssal belt.

Despite their widespread occurrence, very little is known about them; this is because of their elusiveness and the limited reports on them, which is partly due to their scant importance in fisheries. However, from a research point of view, these species represent an exciting group to further assess their ecological roles, morphological and molecular features, and phylogenetic relationships. Indeed, all these topics are underexplored, and many issues are unclear or not well explored. For these reasons, this review manuscript aimed to clarify the Mediterranean Sea distribution of the entire Lampriformes order, provide researchers in this area with a comprehensive reference to support their studies, and enhance the importance of deepening it. The present review summarized what is known on this topic, from the first species descriptions in the Mediterranean basin.

This study was affected by fragmentation of reports, which were often treated superficially and kept in the margins of more extensive studies on species that were deemed commercially more important. Moreover, the phylogenetic positions and relationships within the order have changed many times throughout history, making investigation more difficult. Only recently, with the increased use of molecular approaches to support phylogenies, we have begun to understand these aspects more appropriately.

We showed that four families of this rather vast order are represented in the Mediterranean Sea, of which there are six distributed worldwide. Indeed, Lampridae, Lophotidae, Regalecidae, and Trachipteridae have been reported on since the early 1800s in several areas of the Mediterranean basin. The Lampridae family is represented by the species *L. guttatus*, which is primarily present in the northern area of the central Mediterranean Sea, from both the French and Italian parts of the Ligurian Sea and the Adriatic Sea. The family Lophotidae is, in the same manner, represented by only one species, *L. lacepede*, which has been historically present in Aegean waters and secondly in the Adriatic Sea, while recently occurring in some other areas of the basin. Indeed, this family shows widespread records in the Mediterranean basin, from the Gibraltar Strait to Turkish waters. The Regalecidae family is represented by *R. glesne,* with an evident prevalence of records from the central part of the basin (Ligurian Sea) such as Lampridae, even if supported by a lesser amount of data in this case. The Trachipteridae family is the most abundant and widely distributed taxon among Lampriformes fishes in the Mediterranean Sea. Indeed, it is represented by three species, *T. arcticus*, *T. trachypterus*, and *Z. cristatus*. Regarding the first species, its occurrence has been recorded once in the basin, in the Spanish waters near the Gibraltar Strait, probably representing a specimen that entered from the nearby Atlantic Ocean, where it is more common. On the contrary, *T. trachypterus* and *Z. cristatus* are the most represented species in the Mediterranean Sea, showing a preference for the central part of the basin, with a relatively high number of records from the Adriatic Sea, Ligurian Sea, and the whole Tyrrhenian Sea. The families Radiicephalidae and Veliferidae are not currently reported to be in the Mediterranean Sea.

In the Mediterranean basin, the occurrence of these fishes is mainly linked to occasional capture by longline and midwater trawl fisheries as by-catch. A considerable amount of potential data is lost every day, as these specimens are often thrown back into the sea without further consideration. This reflects what happens in the rest of the world’s seas, where these species are considered rare but not endangered, and all are listed as Least Concern (LC) by the IUCN Red List [238]. In fact, the limited information about them does not permit an adequate evaluation of the conservation status of these species.

Moreover, specimens caught with trawling nets often show very morphologically altered features and are difficult to identify adequately. From this perspective, it is even more essential to use molecular approaches to identify phenotypically similar fish, and to distinguish among them. Moreover, identification keys have changed many times, such as their classification; for this reason, this review did not give particular attention to older, undetailed identifications or personal communications. Current citizen science, supported by appropriate photographic material, is a resource that should be exploited more in studying rare species such as Lampriformes. In any case, without the support of research, it will not be possible to obtain better knowledge. It is necessary for the scientific community to make a more significant efforts, with dedicated projects to deepen the study of this interesting order.

## Figures and Tables

**Table 1:** Summary table of the currently accepted 6 families, 11 genera and 27 species of the Lampriformes order.

Family	Genus	Species
Lampridae (Gill 1862)	*Lampris* (Retzius, 1799)	*Lampris australensis* (Underkoffler, Luers, Hyde and Craig, 2018)
		*Lampris guttatus* (Brünnich, 1788)
		*Lampris immaculatus* (Gilchrist, 1904)
		*Lampris incognitus* (Underkoffler, Luers, Hyde and Craig, 2018)
		*Lampris megalopsis* (Underkoffler, Luers, Hyde and Craig, 2018)
Lophotidae (Bonaparte, 1845)	*Eumecichthys* (Regan, 1907)	*Eumecichthys fiski* (Günther, 1890)
	*Lophotus* (Giorna, 1809)	*Lophotus capellei* (Temminck and Schlegel, 1845)
		*Lophotus guntheri* (Johnston, 1883),
		*Lophotus lacepede* (Giorna, 1809),
		*Lophotus machadoi* (Miranda Ribeiro, 1927)
Radiicephalidae (Osorio, 1917)	*Radiicephalus* (Osório, 1917)	*Radiicephalus elongatus* (Osório, 1917)
		*Radiicephalus kessinger* (Koeda and Ho, 2018)
Regalecidae (Gill, 1884)	*Agrostichthys* (Phillipps, 1924)	*Agrostichthys parkeri* (Benham, 1904)
	*Regalecus* (Ascanius, 1772)	*Regalecus glesne* (Ascanius, 1772)
		*Regalecus russellii* (Cuvier, 1816)
Trachipteridae (Swainson, 1839)	*Desmodema* (Walters and Fitch, 1960)	*Desmodema lorum* (Rosenblatt and Butler, 1977)
		*Desmodema polystictum* (Ogilby, 1898)
	*Trachipterus* (Goüan, 1770)	*Trachipterus altivelis* (Kner, 1859)
		*Trachipterus arcticus* (Brünnich, 1788)
		*Trachipterus fukuzakii* (Fitch, 1964)
		*Trachipterus ishikawae* (Jordan and Snyder, 1901)
		*Trachipterus jacksonensis* (Ramsay, 1881)
		*Trachipterus trachypterus* (Gmelin, 1789)
	*Zu* (Walters and Fitch, 1960)	*Zu cristatus* (Bonelli, 1819)
		*Zu elongatus* (Heemstra and Kannemeyer, 1984)
Veliferidae (Bleeker, 1859)	*Metavelifer* (Walters, 1960)	*Metavelifer multiradiatus* (Regan, 1907)
	*Velifer* (Temminck and Schlegel, 1850)	*Velifer hypselopterus* (Bleeker, 1879)

**Table 2:** Bibliographic references of the Lampridae family records in the Mediterranean Sea.

Family, Genus	Species	Year	Mediterranean Area	Number of Specimens	References
Lampridae, *Lampris*	*Lampris guttatus*	before 1807	Ligurian Sea (Italy)	1	Spinola (1807)
		before 1826	Nice (France)	1	Risso (1826)
		1829	Toulon (France)	1	Cuvier and Valenciennes (1835)
		1829	Marseille (France)	1	Cuvier and Valenciennes (1835)
		1898	Viareggio (Italy)	1	Ariola (1904)
		1901	Camogli (Italy)	1	Ariola (1904)
		1902	Novigrad Sea (Croatia)	2	Katurić (1902)
		1956	Bakar Bay (Croatia)	1	Crnković (1957)
		before 1970	Finale Ligure (Italy)	1	Tortonese (1970)
		1974	Camogli (Italy)	1	Cattaneo and Bava (2009)
		1979	Pizzo (Italy)	1	Andaloro and Di Natale (1979)
		1983	Unknown	1	Parin and Kukuyew (1983)
		1994	Neretva estuary (Croatia)	1	Bartulović (in Dulčić et al. 2005)
		1997	Toulon/Embiez (France)	1	Francour et al. (2010)
		1997	Bandol (France)	1	Francour et al. (2010)
		1998	Anzio (Italy)	3	Psomadakis et al. (2006)
		2000	Porquerolles Island (France)	1	Francour et al. (2010)
		2000	Sète (France)	1	Francour et al. (2010)
		2001	Anzio (Italy)	1	Psomadakis et al. (2006)
		2002	Nea Skioni (Greece)	1	Sinis (2004)
		2003	Embiez (France)	1	Francour et al. (2010)
		2003	Vir Island (Croatia)	1	Dulčić et al. (2005)
		2004	Bormes-les-Mimosas (France)	2	Francour et al. (2010)
		2007	Antibes (France)	2	Francour et al. (2010)
		2007	Embiez (France)	1	Francour et al. (2010)
		2007	Marseille (France)	1	Francour et al. (2010)
		2007	North Bastia (France)	1	Francour et al. (2010)
		2008	Antibes (France)	1	Francour et al. (2010)
		2008	Giens (France)	1	Francour et al. (2010)
		2008	off Le Levant Island (France)	1	Francour et al. (2010)
		2008	Karpathos Island (Greece)	1	Corsini-Foka (2009)
		2008	off Nice (France)	2	Francour et al. (2010)
		2008	Porquerolles Island (France)	1	Francour et al. (2010)
		2008	off Cagnes sur Mer (France)	1	Francour et al. (2010)
		2008	Saint-Raphaël (France)	2	Francour et al. (2010)
		2008	Gouraya (Algeria)	1	Francour et al. (2010)
		2008	Camogli (Italy)	3	Francour et al. (2010)
		2009	Camogli (Italy)	1	Francour et al. (2010)
		2009	Cannes (France)	1	Francour et al. (2010)
		2009	East Corsica (France)	1	Francour et al. (2010)
		2009	Radhima, Vlora Bay (Albania)	1	Bego and Kashta (2012)
		2012	Mali Ston Bay (Croatia)	1	Šprem et al. (2014)
		2017	off Erdemli coast (Turkey)	1	Ergüden et al. (2019)
		2021	Ghar El Melh (Tunisia)	1	Ennajar et al. (2020)

**Table 3:** Bibliographic references of the Lophotidae family records in the Mediterranean Sea. N.R. means not reported.

Family, Genus	Species	Year	Mediterranean Area	Number of Specimens	References
Lophotidae, *Lophotus*	*Lophotus lacepede*	before 1890	Adriatic Sea (Croatia)	1	Kolombatović (1890)
		before 1948	North Adriatic Sea	N.R.	Soljan (1948)
		before 1950	Adriatic Sea	1	Morović (1950)
		1970	Strait of Messina (Italy)	1	Tortonese (1970)
		before 1970	Genova (Italy)	1	Tortonese (1970)
		1971	Strait of Messina (Italy)	1	Magazzù and Zaccone (1971)
		1979	Sitges (Spain)	1	Portas and Del Cerro (1979)
		1980	Strait of Gibraltar	1	Ray (1983)
		1980	Strait of Messina (Italy)	1	Giuffrè et al. (1980)
		before 1986	Ionian Sea	N.R.	Palmer (1986)
		before 1987	Central Mediterranean	N.R.	Bauchot (1987)
		before 1988	Greek Sea	N.R.	Papacostantinou (1988)
		1989	Sitia, Crete Island (Greece)	1	Minos et al. (2015)
		1999	Ischia Island (Italy)	1	Bussotti et al. (1999)
		2001	Gökova Bay (Turkey)	1	Bilecenoglu et al. (2001)
		2002	Calabria (Italy)	1	Tripepi et al. (2004)
		2003	Souda Bay, Crete Island (Greece)	1	Minos et al. (2015)
		2005	Balearic region (Spain)	1	Rodriguez et al. (2013)
		2007	North Adriatic Sea (Croatia)	2	Dulčic and Ahnelt (2007)
		2008	North Adriatic Sea (Croatia)	1	Dulčic and Soldo (2008)
		2011	Southern Adriatic (Croatia)	1	Sprem et al. (2014)
		2011	cape Poseidi (Greece)	1	Minos et al. (2015)
		2012	Punta Licosa (Italy)	1	Psomadakis et al. (2012)
		2015	San Vito Lo Capo (Italy)	1	Falsone et al. (2017)
		2016	Aegean Sea (Greece)	1	Aga-Spyridopoulou et al. (2019)
		2017	northern Aegean Sea (Turkey)	1	Dalyan et al. (2021)
		2017	Aegean Sea (Greece)	3	Aga-Spyridopoulou et al. (2019)
		2018	Aegean Sea (Greece)	1	Aga-Spyridopoulou et al. (2019)
		2018	Çanakkale (Turkey)	1	Tunçer and Kanat (2019)
		2019	Levantine Sea (Turkey)	1	Yapici (2019)
		2021	Ammouliani Island (Greece)	1	Minasidis and Kaminas (2021)
		2021	off Banias (Syria)	1	Ali et al. (2021)

**Table 4:** Bibliographic references of the Regalecidae family records in the Mediterranean Sea. N.R. means not reported.

Family, Genus	Species	Year	Mediterranean Area	Number of Specimens	References
Regalecidae, *Regalecus*	*Regalecus glesne*	1826	Nice (France)	2	Risso (1826)
		1830	Nice (France)	3	Cuvier and Valenciennes (1835)
		1877	Nice (France)	1	Giglioli (1880)
		1891	Isola d’Elba (Italy)	1	Damiani (1918)
		1897	Beaulieu saint-Jean (Francia)	1	Vayssiére (1917)
		1903	Noli (Italy)	1	Ariola (1904)
		1906	Borghetto S. Spirito (Italy)	1	Vinciguerra (1918)
		1908	Arenzano (Italy)	1	Vinciguerra (1918)
		1910	Monaco (France)	1	Vayssiére (1917)
		1913	Castiglioncello (Italy)	1	Vinciguerra (1918)
		1915	Albissola (Italy)	1	Vinciguerra (1918)
		1917	S Margherita Ligure (Italy)	1	Vinciguerra (1918)
		1932	Rimini (Italy)	1	Padovani (1933)
		1950	Genova (Italy)	1	Guiglia (1950)
		1969	Mazzarrón (Spain)	1	Lozano-Cabo (1969)
		before 1970	Ligurian Sea	N.R.	Tortonese (1970)
		before 1971	Aegean Sea	N.R.	Ondrias (1971)
		1974	Olivieri (Italy)	3	Berdar et al. (1975)
		1980	Strait of Messina (Italy)	1	Cavallaro et al. (1980)
		before 1988	Aegean Sea	N.R.	Papacostantinou (1988)
		1993	Gennadi (Greece)	1	Corsini-Foka (2009)
		2002	Marseille (France)	2	Quero et al. (2003)
		2003	Arenzano (Italy)	1	Psomadakis et al. (2008)
		2009	Stobrec (Croatia)	1	Dulčic et al. (2009)
		2010	Palagruža Island (Croatia)	1	Dragičević et al. (2011)
		2012	Arenzano (Italy)	1	Psomadakis et al. (2012)
		2012	Terracina (Italy)	1	Psomadakis et al. (2012)

**Table 5:** Bibliographic references of the Trachipteridae family records in the Mediterranean Sea. N.R. means not reported. * The number outside the brackets indicates the collected specimens; the number inside the brackets indicates the observed specimens.

Family, Genus	Species	Year	Mediterranean Area	Number of Specimens	References
Trachipteridae, *Trachipterus*	*Trachipterus arcticus*	before 1986	Spanish coast	1	Robins and Ray (1986)
Trachipteridae, *Trachipterus*	*Trachipterus trachypterus*	1881	Adriatic Sea (Croatia)	N.R.	Kolombatović (1890)
		1888	Gulf of Trieste (Italy)	2	Marcuzzi (1972)
		before 1980	Grignano (Italy)	46	Jardas (1980)
		1980	Off Sardinian coast (Italy)	3	Cau (1980)
		1992	Grignano (Italy)	1	Bussani (1992)
		1992	Ronek Cape (Slovenia)	1	Dulčic and Lipej (1997)
		1996	Eastern Levant Sea (Israel)	1	Golani (1996)
		1996	Stončica (Croatia)	1	Dulčic (1996)
		2000	Anzio (Italy)	1	Psomadakis et al. (2006)
		2006	Gulf of Trieste (Italy)	5	Borme and Voltolina (2006)
		2010	Cephalonia Island (Greece)	1	Mytilineou et al. (2013)
		2010–2013	Ligurian Sea (Italy)	N.R.	Garibaldi (2015)
		2016	Çeşme (Turkey)	1	Yapici (2019)
		2017	Scanzano Ionico (Italy)	1	Tirlongo et al. (2019)
		2018	Porto Cesareo (Italy)	1	Tirlongo et al. (2019)
		2018	Marzamemi (Italy)	1	Tirlongo et al. (2019)
		2018	Izola (Slovenia)	1	Lipej et al. (2018)
		2018	Ponza (Italy)	4 (18) *	Macali et al. (2020)
		2019	Pianosa (Italy)	1	Tiralongo et al. (2020)
		2020	Gulf of Antalya (Turkey)	1	Gökoğlu and Özen (2021)
		2021	Maliakos Gulf (Greece)	1	Kaminas et al. (2021)
		2021	Pagasitikos Gulf (Greece)	1	Kaminas et al. (2021)
		2021	Attica Peninsula (Greece)	2	Kaminas et al. (2021)
		2021	Kerkira Island (Greece)	2	Kaminas et al. (2021)
Trachipteridae, *Zu*	*Zu cristatus*	1918	La Spezia (Italy)	1	Bonelli (1920)
		1954	Gulf of Tunis (Tunis)	1	Postel (1955)
		1955	Palma de Mallorca (Spain)	1	Oliver (1955)
		1958	Gulf of Genova (Italy)	1	Tortonese (1958)
		1969	Blanes (Spain)	1	Ibáñez and Gállego (1974)
		1976	Gulf of Genova (Italy)	1	Gavagnin (1976)
		1979	Off Sardinian coast (Italy)	1	Cau (1980)
		before 1980	Central/northern Adriatic Sea	16	Jardas (1980)
		1980	Arenys de Mar (Spain)	1	Roig and Demestre (1982)
		1981	Malgrat de Mar (Spain)	1	Roig and Demestre (1982)
		1998	Gulf of Castellammare (Italy)	2	Bianco et al. (2006)
		1998–2000	Duće (Croatia)	eggs	Dulčic (2002)
		2000	Anzio (Italy)	1	Psomadakis et al. (2006)
		2003	Gulf of Genova (Italy)	2	Psomadakis et al. (2007)
		2004	Vis Island (Croatia)	1	Dulčic et al. (2014)
		2009	Zadar (Croatia)	1	Dulčic et al. (2014)
		2009	Mahdia (Tunis)	1	Bradai and El Ouaer (2012)
		2010–2013	Ligurian Sea (Italy)	N.R.	Garibaldi (2015)
		2013	Isla de Cabrera (Spain)	1	García–Barcelona et al. (2014)
		2013	Hvar Island (Croatia)	1	Dulčic et al. (2014)
		2014	Isla de Formentera (Spain)	1	García–Barcelona et al. (2014)
		2014	Vibo Valentia (Italy)	1	Zenetos et al. (2015)
		2015	San Vito lo Capo (Italy)	1	Fasone et al. (2017)
		2017	Civitavecchia (Italy)	1	Tiralongo et al. (2019)
		2018	Ponza (Italy)	1	Tiralongo et al. (2019)
		2018	Avola (Italy)	1	Tiralongo et al. (2019)
		2018	Porto Cesareo (Italy)	2	Tiralongo et al. (2019)
		2018	Briatico (Italy)	1	Tiralongo et al. (2020)
		2019	Pianosa (Italy)	1	Tiralongo et al. (2020)
		2019	Capraia (Italy)	1	Tiralongo et al. (2020)
		2019	Fiumicino (Italy)	1	Tiralongo et al. (2020)
		2021	Dodecanese Islands (Greece)	1	Kaminas et al. (2021)
		2021	Lesvos Island (Greece)	1	Kaminas et al. (2021)

## Data Availability

Not applicable.

## Supplementary Materials

The following supporting information can be downloaded at: https://www.mdpi.com/article/10.3390/biology11101534/s1, Table S1: Coding sequences of mt-COI from 21 Lampriformes species currently available on NCBI database used in our phylogenetic reconstruction of Lampriformes relationships.
