# Supplementary material for: Distribution of the Order Lampriformes in the Mediterranean Sea with Notes on Their Biology, Morphology, and Taxonomy
Source: Biology (Basel). 2022 Oct 19;11(10):1534. doi: 10.3390/biology11101534 (PMC9598601; doi:10.3390/biology11101534)
Supplement: Supplementary file 1 [file biology-11-01534-s001.zip › biology-1942506-supplementary.pdf]

Supplementary Table S1. Coding sequences of mt-COI from 21 Lampriformes species currently available on NCBI database used in our phylogenetic reconstruction of Lampriformes relationships.

GenBank Accession ID	Scientific name	COI length (bp)
MN123259.1	*Agrostichthys parkeri*	648
GU440303.1	*Desmodema lorum*	652
MN117725.1	*Desmodema polystictum*	658
BBG74528.1	*Eumecichthys fiski*	516
JF931925.1	*Lampris australensis*	655
GU992944.1	*Lampris guttatus*	662
MN123398.1	*Lampris immaculatus*	645
JF931946.1	*Lampris incognitus*	655
LC521832.1	*Lampris megalopsis*	685
MN123409.1	*Lophotus cappellei*	645
KR086866.1	*Lophotus lacepede*	665
EU366588.1	*Metavelifer multiradiatus*	782
MN123480.1	*Regalecus glesne*	645
KU943114.1	*Regalecus russelii*	552
GU440558.1	*Trachipterus altivelis*	652
MT323519.1	*Trachipterus arcticus*	629
HQ564197.1	*Trachipterus jacksonensis*	652
OM527150.1	*Trachipterus trachypterus*	652
GU673640.1	*Velifer hypselopterus*	652
MT323474.1	*Zu cristatus*	632
MN123529.1	*Zu elongatus*	651
